# Time to re‐envisage integrity among nurse leaders

**DOI:** 10.1111/jonm.13557

**Published:** 2022-02-15

**Authors:** Kathleen Markey, Mairead Moloney, Owen Doody, Simon Robinson

**Affiliations:** ^1^ Department of Nursing and Midwifery, Faculty of Education and Health Sciences, Health Research Institute University of Limerick Limerick Ireland; ^2^ Department of Nursing and Midwifery, Faculty of Education and Health Sciences University of Limerick Limerick Ireland; ^3^ Leeds Beckett University Leeds UK; ^4^ University of Leeds Leeds UK

**Keywords:** courage, dialogue, honest, integrity, nurse leadership

## Abstract

**Aim:**

This paper highlights integrity as a central tenet in the journey of ethical leadership among nurse leaders and dialogue as a way of working within integrity.

**Background:**

Nurse leaders play a critical role in ensuring ethically sound, safe patient care by supporting staff and fostering positive working environments. Although there is an abundance of literature on leadership, no universally accepted leadership theory exists. Hence, it can be difficult to apply leadership theory and principals to real‐life clinical practice.

**Evaluation:**

From the literature, it is evident that integrity is a crucial aspect of leadership. This paper proposes suggestions for nurturing integrity and fostering open and honest dialogue.

**Key issues:**

Globally, public health care is complex and evolving and effective nursing leadership is paramount to meet public health needs and support health care systems.

**Conclusion:**

This paper explores integrity with leadership, re‐envisaging personal and professional integrity as a portal to authentic leadership, which has human relationships and dialogue at its core.

**Implications for Nursing Management:**

Nurse leaders need support in guiding the nursing profession and promoting ethically sound patient care. The true nature of leadership is dialogue, and nurturing a culture of listening and openness at different levels within an organisation is crucial.

## 1 INTRODUCTION

In an increasingly multifaceted, uncertain and evolving health care systems, the need for resilient nurse leaders who can respond to expanding demands in innovative, ethical and solution focussed ways is paramount. The recent global COVID‐19 health care crisis illuminated multiple deep underlying problems within health care organisations that were unaddressed and highlighted their lack of preparedness to manage in a crisis. The lack of personal, professional and organisational learning from previous health care failings perhaps contributed to the lack of preparedness for the crisis. Continuing suggestions of deficient standards of nursing care (Chaboyer et al., 2021) suggest systematic failures that remain unresolved and highlight some of the stark realities facing nurse leaders.

In an era of evolving health care services and challenging environments, the need for effective leadership is crucial. Recognition needs to be given to the growing demands on nurses and nurse leaders in busy and complex environments, which are intensifying at a rapid pace in a system that is slow to respond, adapt and learn lessons (Wills et al., 2015). Krichbaum et al. (2007) refer to this phenomenon as ‘complexity compression’, which is a term used to illuminate the additional and sometimes unplanned responsibilities on top of normal responsibilities leading to impossible circumstances if the appropriate action is not considered. Consequently, it is time to think differently about leadership and this calls for new ways of navigating leadership within increasingly complex health care systems, to adapt more speedily in ethically sound ways. One way to do this is by re‐envisaging integrity, a crucial aspect of leadership, which is often discussed but rarely defined. This paper considers integrity a central tenet in the journey of ethical leadership and dialogue as a philosophy and a way of working within integrity where leaders manage both society of mind and society of members through a reciprocal exchange.

## 2 LEADING WITH INTEGRITY

Nurse leaders play a critical role in ensuring the delivery of safe, compassionate and quality patient care through supporting staff (Markey et al., 2020) and fostering positive working environments (Kim et al., 2018). Effective leaders are charismatic, inspirational and have integrity and consideration for the needs of individuals (Copeland, 2016). Although there is an abundance of literature on leadership concepts and theories, no universally accepted leadership theory exists. Harris and Mayo (2018) draw many comparisons on various leadership theories and models showing their evolution and also highlighting how different approaches work in different contexts. Historically, nursing leadership was rooted in hierarchical authoritarian styles (Doody & Doody, 2012), which conjured a following of people with unwavering respect and unquestioning of authority. With respect to nursing history, unpeeling from this hierarchical cultural matrix is sometimes difficult. Transformational leadership (Wang et al., 2018), ethical leadership (Markey et al., 2021), authentic leadership (Avolio & Gardner, 2005) and complexity leadership (Khan et al., 2018) have dominated the nursing literature. Although these leadership theories come from different philosophies, they are all grounded in positive relational styles and focus on human relationships to promote positive change. However, implementing these theories in daily leadership practice remains difficult and abstract (Harris & Mayo, 2018).

One way to re‐envisage leadership is to explore integrity, a common thread within leadership literature (Bauman, 2013). Despite many different meanings of integrity having been offered, the focus is on identity, taking responsibility and owning one's history and character expressed in one's commitments (to purpose, ideals, values, goals, projects) to one's reputation (Robinson, 2016). This offers a view of integrity that is holistic and interpersonal, involving ongoing learning and creative response. To function with integrity, one's words, thoughts and actions must engage morally, emotionally, psychologically and spiritually. This suggests that integrity can be viewed as a journey of learning and growth and a journey of continuously realigning oneself with core purpose and values. Hence, Western (2008) writes about the ‘formation’ of leaders. This process involves engaging complex identities, including nurse, nurse leader, male/female and parent. Moreover, integrity at all levels (individual, group and organisation) is codependent and can never be mutually exclusive (Palanski & Yammarino, 2007). This suggests that integrity is cocreated at multilevels of an organisation and taking ownership of that cocreation is where leaders awakening and growth lies.

Integrity thus is focused on critical agency (reflecting critically, basing professional judgements on core purpose and ethical values), mutual accountability (within and beyond the profession) and shared responsibility for creatively embodying the core professional purposes. It is embodied in attention to narrative building, critical deliberation and dialogue, enabling the practice of interrelated personal, professional, organisational and interorganisational responsibility (Robinson & Doody, 2021).

Much has been written about integrity as a virtue (moral disposition or capacity), ranging from a discrete virtue (Curzer, 2014) to an epistemic virtue, enabling perceptual distance (Scherkoske, 2013), to a ‘mega‐virtue’ which connects all the other virtues (Solomon, 2008). The last of these suggests that integrity is the capacity (virtue) to take responsibility for critical reflection on identity and practice and that this interrelates with all the holistic virtues: moral (courage, fairness, respect, honesty etc.), psychological/affective (empathy), intellectual (practical wisdom) and practical (competency). Palanski et al. (2015) provide an example of this interrelationship, indicating courage and integrity as intertwined in effective leadership. Courage is needed to question perceived integrity at various levels, and leaders who critically review the beliefs and values underpinning their leadership approaches are more likely to be courageous. Courage involves honesty (candour) about actions and omissions, being able to admit when you have made poor decisions and have the resilience (psychological virtue) to question oneself and others. However, the impact of organisational processes and hierarchical management structures can negatively impact integrity and with that courageous behaviour (Pajakoski et al., 2021). Management that focuses on narrow targets and related isomorphism aim to determine the behaviours of nurses (Robinson & Doody, 2021) and thus constrain nurse leaders' ability to fulfil their role. The emphasis is precisely on unthinking practice with professional purpose uncritically assumed (Roberts & Ion, 2015). Dialogue in this light comes to be seen as an expression of conflict, and thus as a threat both to trust and success, rather than an embodiment of shared support and creativity, actually enabling trust. Hence, the need to nurture courageous leaders to initiate dialogue at various levels becomes critical, at board level, in teams, between individual colleagues and between professions. It takes courage to examine integrity at an individual level while remaining open to the perspectives of the wider team and the institution. It takes courage to push boundaries, become a pattern disrupter, respond to criticisms evenly, and even greater courage to recognize one's own biases and failings as a leader. Importantly, this recognizes that no leadership is free from these problems, and no system or institutional culture can obviate the need to practice such virtues, and thus, any cultural change should focus on how to nurture them (Moore, 2012). Integrity is at the heart of leadership (Sahraei Beiranvand et al., 2021), and leaders need to own the chasm between how things have become and how they should be, to move forward individually and collectively.

## 3 FOSTERING DIALOGUE

To begin moving with integrity, nurturing a culture of listening and open dialogue at the different levels within an organisation is crucial (Macnamara, 2015). The true nature of leadership with integrity is dialogue, which forms a complex web of lived experience (personal, professional and cultural) which the leader continuously engages (consciously and unconsciously). It is the sum of the individual's lived experiences that determines how an individual leader at a specific time, place and situation will respond or choose to act. No one leadership ‘technique’ or ‘style’ can fully inform an individual leaders' actions. Yet, it is sometimes difficult to be a catalyst of change and approach dialogue with others in a way that is perceived as open, meaningful and respectful rather than as a denunciation of power and thus a threat to leadership. However, professional agency can only be developed through respectfully engaging difference and its associated affective dissonance and cognitive challenge (both critical to learning).

Dialogue is also ontological (Bakhtin, 1984) involving the encounter between individuals, the profession or institution. Each then becomes responsible for the worth and meaning of that world, naming it. Avoiding dialogue narrows the perspective, values and relational awareness of leadership, leading in many cases to exclusion and dehumanization of the stakeholders (Freire, 1972), including fellow professionals, patients and patients' families (Francis, 2013). Dialogic leadership is both a philosophy and a way of working where the leader manages both the society of mind and the society of members through a reciprocal exchange. A leader needs to create a constructive thought pattern within their team by establishing a team‐thinking and problem‐solving mechanism that is team‐based. This will offset groupthink syndrome, as the team becomes a dynamic living system with potentials for creative ideas and can perform beyond expectations. Dialogue in this process is unrehearsed, stressing genuine accountability and openness to ideas, concerns and people. Arguments against this are dominated by the belief that there is not enough time for such engagement. This view of time, however, is based on linear thinking and limited narrow deadlines (Gardiner et al., 2021). In any case, the attention to dialogue can ensure both shared responsibility and positive engagement with dissonance, without which the professional culture can lose focus and subsequently develop a negative culture (Francis, 2013). At organisation level, dialogue and deliberation could focus on the different perspectives of core purpose and principles, not simply achieving narrow objectives, embodying the virtues of governance (Moore, 2012).

Dialogic leadership enables the leader to adapt and reflect changing circumstances remaining focused in personal/professional identity, and thus their core shared worth, and related ethical principles. The future depends partly on leaders' ability to find intelligent and imaginative solutions to complex and difficult issues and create the conditions for dialogue in the context of the organisation, profession, team and society. Isaacs (1999) suggests four qualities/abilities needed to support this dialogue leadership: (1) to evoke people's genuine voices, (2) to listen deeply, (3) to hold space for and respect as legitimate other people's views and (4) to broaden awareness and perspective. Nurse leaders need to balance the embodying of all these qualities while enabling them in others. However, this is not without its challenges. Nurse leaders need to nurture inclusive and inviting spaces that bring team members together to openly think, talk and share ideas about problems and solutions in a reciprocal (iterative) learning environment. Incorporating Kantor's (2012) four‐player model (move, follow, oppose and bystand) is a framework that can guide meaningful dialogue and nurture cross‐fertilization of ideas and shared sense‐making. Applying system theory to Kantor's (2012) four‐player model, an effective team is one that works with each of the four players reinforcing each other's role, as domination by any player can hamper or undermine the performance of the team. Therefore, through dialogue, leaders maintain a healthy atmosphere of divergence by creating an environment of trust and openness, where team members speak up and critique each other's ideas and opinions without fear. A leader having a planning or brainstorming session, here they first assume a mover stance, acting as a host and stage‐setter. After outlining the purpose and focus of the meeting, the leader may then shift into the bystander stance. Thus, creating the stage for engagement of team members to occur where the conversation proceeds, opinions and ideas are raised. By operating as a bystander, the leader can observe others' participation and watch for tensions that may exist and how effectively the team manages collective decision making. During the process, the leader can move to the opposer stance to ask critical questions to focus the discussion and ensure coverage of key aspects. As the discussion progresses, the leader may assume a follower stance to signal agreement with what others have said, progress discussion momentum and summarize the discussion and priorities that have surfaced. When the discussion has run its course, the leader then reverts to a bystander stance to make general process comments and identify if further discussion is needed. Finally, the leader returns to a mover stance to summarize the work the group has done and to suggest consensus on a topic has been reached and that the team is ready to act.

## 4 CONCLUSION

This paper illuminates the importance of re‐envisaging integrity, as a central tenet in the journey of effective leadership in increasingly complex health care environments and dialogue as a way of integrating ethical principles, creative practice and holistic virtues, focused on shared responsibility.

## 5 IMPLICATIONS FOR NURSE MANAGERS

In an era of evolving health care services and challenging environments, the need for open and honest dialogue at all levels is paramount. Reflection on core purpose can be developed through genuine dialogue across the institution, not simply ‘consultation’ (usually not reciprocal). Team meetings can focus on reflective dialogue, both reinforcing core worth and purpose and enabling the exercise of imagination and creative responses in increasingly complex health care settings. Nurse leaders play a critical role in nurturing dialogue that encourages meaningful critical thinking and collective action steeped in professional purpose and ethical principles. Listening deeply, seeking to consciously understand and responding honestly may be the first steps on the leadership journey, enabled by virtues such as courage, honesty and empathy.

## CONFLICT OF INTEREST

The authors declare no conflict of interest.

## AUTHOR CONTRIBUTIONS

All authors contributed to the drafting and finalizing of the manuscript preparation.

## FUNDING INFORMATION

No specific grant from any funding agency in the public, commercial or not‐for‐profit sectors was received.

## ETHICS STATEMENT

Ethical approval was not required as this is a commentary paper, and no data collection was carried out.

## Data Availability

Data sharing is not applicable to this article as no datasets were generated or analysed during the current study/preparation of this paper.
